# Epicardial Fat Necrosis After COVID-19 Infection: A Case Report

**DOI:** 10.7759/cureus.25154

**Published:** 2022-05-20

**Authors:** Mohamad Zayour, Mahmoud Karaki, Rana Al Ashkar, Elie Chammas

**Affiliations:** 1 Cardiology, University of Balamand, Beirut, LBN; 2 Diabetes and Endocrinology, University of Balamand, Beirut, LBN; 3 Internal Medicine, Faculty of Medicine, Lebanese University, Beirut, LBN; 4 Cardiology, Clemenceau Medical Center, Beirut, LBN

**Keywords:** epipericardial fat necrosis, covid-19, case report, sars-cov-2, epicardial fat necrosis

## Abstract

Epipericardial or epicardial fat necrosis (EFN) is a self-limited inflammatory process occurring in the mediastinal fat surrounding the heart. It is an uncommon cause of acute chest pain and mimics more critical clinical disorders such as acute coronary syndrome, aortic dissection, and pulmonary embolism. However, EFN is frequently overlooked and under-recognized in emergency departments (EDs) owing to the unfamiliarity of this condition among physicians and radiologists. Herein, we present the case of a previously healthy young male patient, with a recent history of mild COVID-19 infection (two weeks before presentation), who presented to the ED for acute chest pain. Paraclinical evaluation including computed tomography (CT) of the chest revealed fat stranding along with the left epicardial fat pad in favor of EFN.

## Introduction

One of the most common causes of emergency department (ED) admissions is chest pain. The latter has a vast differential diagnosis varying from mild to lethal conditions. EFN is an unusual benign cause of chest pain and is often not acknowledged [1]. 

To our knowledge, there are no reports linking COVID-19 infection to epicardial fat necrosis (EFN) in Lebanon. Thus, here we submit a case of EFN in a young male patient with a recent history of COVID-19 infection.

## Case presentation

A 19-year-old male patient, previously healthy, presented to the ED for acute onset chest pain and dyspnea. A review of the systems is significant for smoking history, vaccinated double dose against SARS-CoV-2, with a recent (two weeks prior to presentation) polymerase chain reaction (PCR)-proven COVID-19 infection consisting of generalized fatigue and fever without any associated respiratory symptoms (mild disease course). Family history is not significant for any cardiac disease. The chest pain started a few days before the current presentation, mainly left-sided, radiating to the left shoulder, and pleuritic in nature with no other symptoms.

**Figure 1:**
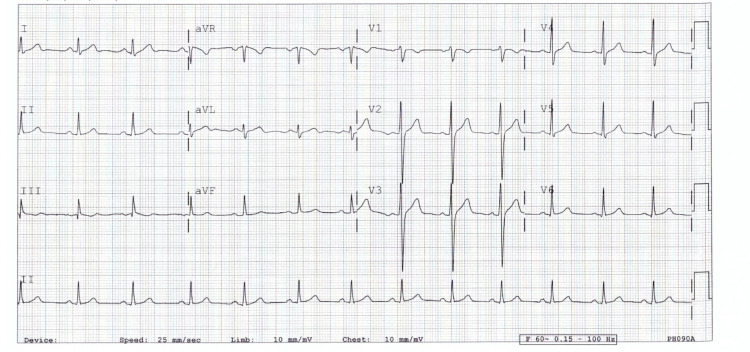
A normal electrocardiogram

Upon ED presentation, he was afebrile, normotensive, not tachycardic nor tachypneic. The patient denied local tenderness and no friction rub was heard during cardiac auscultation. In addition to the laboratory tests, shown in Table 1, troponin was not detectable. Electrocardiogram was performed and showed no abnormalities (Figure 1 and Table 2).

**Table 1:** The initial laboratory results

Test	Value	Normal Range
WBCs	8.97 x10^ 3/microL	
Hemoglobin	13.5 g/dL	
Platelet count	248 000/microL	
Creatinine	0.64	
D-Dimer	188 ng/mL	Normal: <255
C-reactive protein (CRP)	2.834	Normal:<1
Troponin Level	<0.01 ng/mL	

**Table 2:** EKG values reading

Rate	77
PR	144
QRSD	96
QT	376
QIc	426
Axis	
P	30
QRS	68
T	33

**Figure 2:**
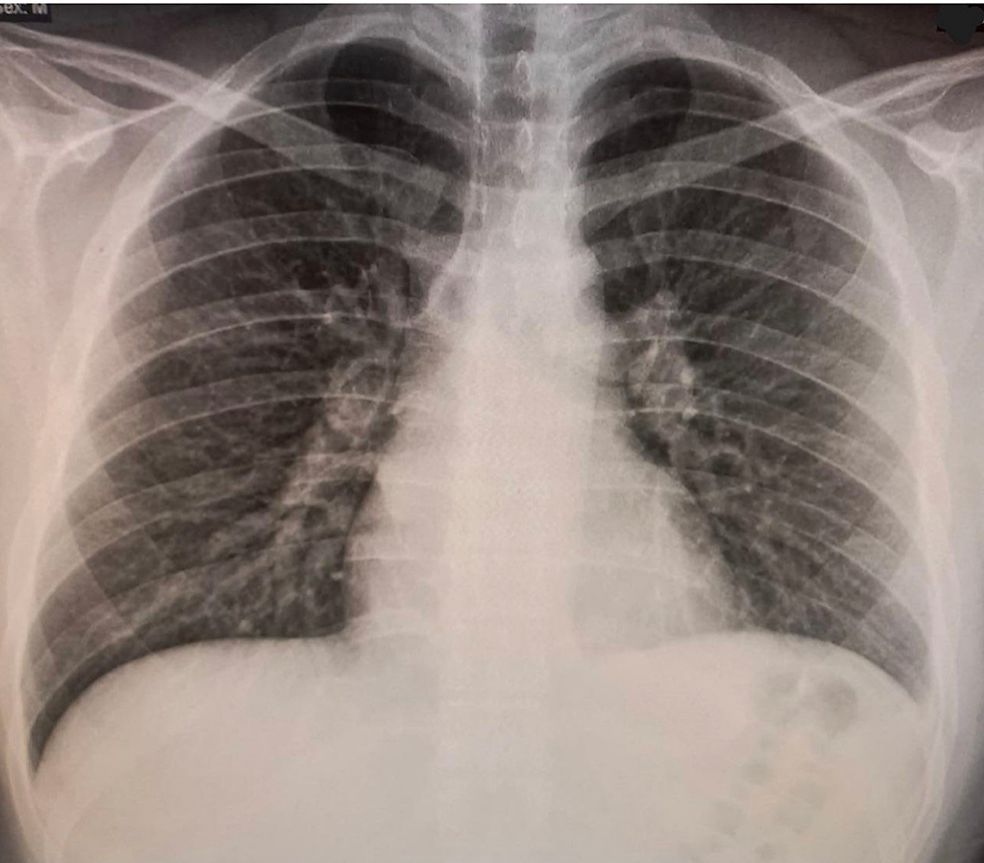
Chest x-ray showing no abnormal findings

**Figure 3:**
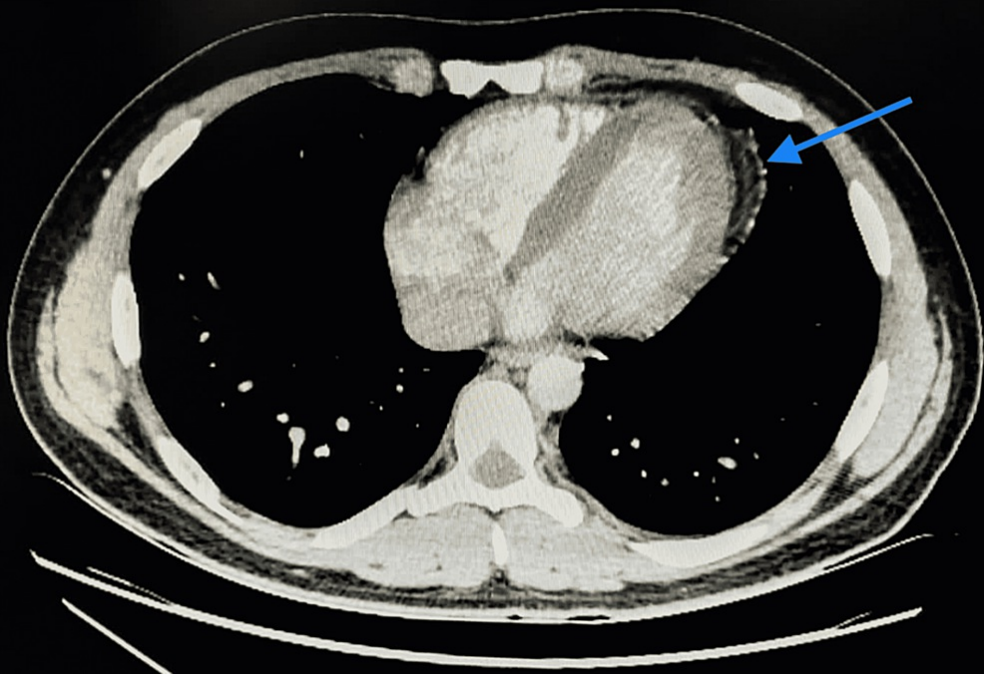
CT angiocoronal view showing pericardial fat stranding (blue arrow).

**Figure 4:**
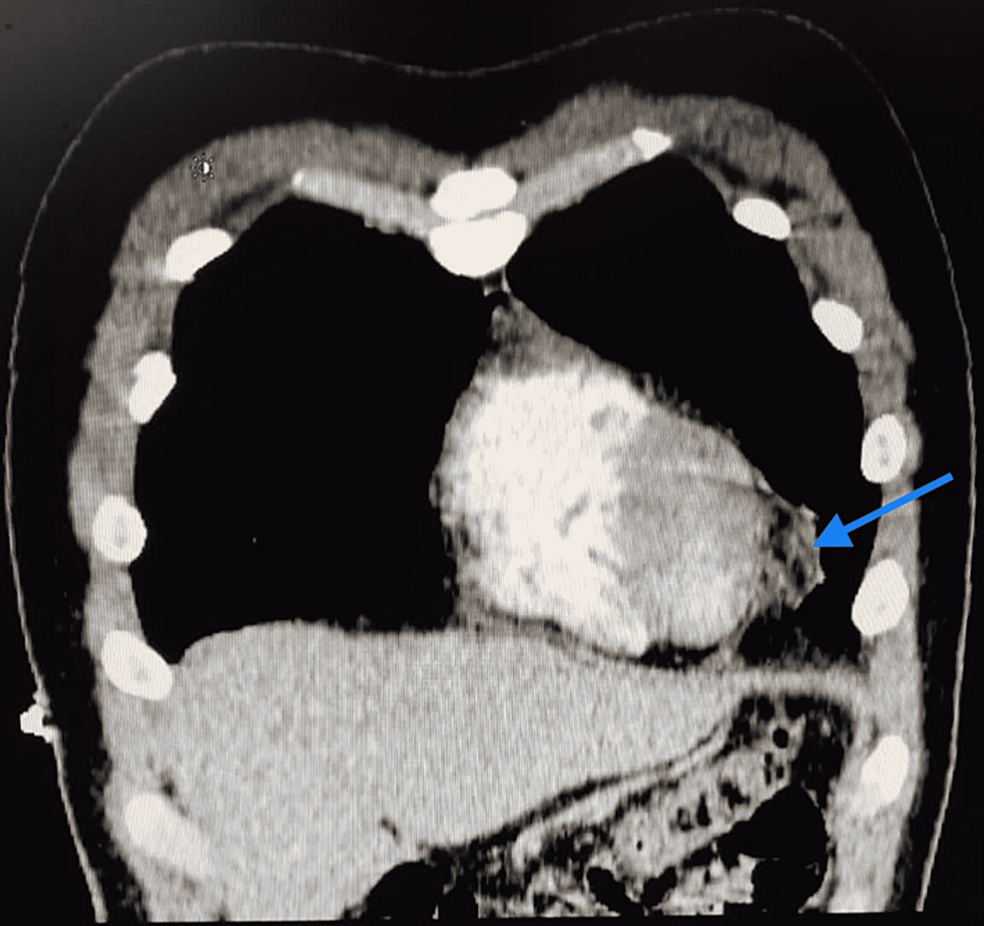
Sagittal view of the pericardial fat stranding (blue Arrow) shown on the CT angio.

Further investigations included a chest x-ray (CXR) that turned out normal (Figure 2). On the following day of admission, C-reactive protein (CRP) and D-dimer trended upward (6,739 mg/dL and 330 ng/mL, respectively). Accordingly, echocardiography was performed with a normal result, and a CT angiography of the chest was done showing no evidence of pulmonary embolism with normal lung parenchyma. However, fat stranding along with the left epipericardial fat pad (Figures 3, 4) was noted suggesting fat necrosis. Before confirming the diagnosis, pericarditis was ruled out by the absence of the following: pericardial effusion on CT, chest pain in the presentation, and that EKG changes.

Hence, the diagnosis of EFN secondary to COVID-19infection was made. The patient was discharged on colchicine and non-steroidal anti-inflammatory drugs with subsequent improvement of symptoms observed during his follow-up appointment after one week of discharge.

## Discussion

EFN is an unusual benign disease initially portrayed in 1957 by Jackson et al. [2,3]. The common presentation is acute pleuritic chest pain that can mimic other serious diseases [4].

The actual rate of this disorder is unspecified because of its limited events. However, there has been a growth in the documentation of EFN in recent years, possibly due to the better imaging modalities, thus the incidence is estimated at 2.2% in patients who underwent chest CT for chest pain in the ED [5]. Although EFN is considered an inflammatory process that takes place within the epipericardial fat leading to encapsulated necrosis, similarly to fat necrosis in breast tissue after injury, peripancreatic fat in pancreatitis and epiploic appendagitis [1,6], the exact pathophysiology is not well established with some suggested mechanisms still under investigations.

SARS-CoV-2 being known as a respiratory causing illness did not have a fully explained mechanism of action (ACE2 moderated direct myocardial damage, hypoxia-induced destruction, microvascular injury, and SIRS) to elaborate its effect on other organs. It is known to have caused a wide range of clinical conditions from asymptomatic to respiratory failure. However, it also appeared to have had an important effect on the cardiac system. Those cardiac manifestations were varying from heart failure to arrhythmias, myo/pericarditis, and even sudden cardiac death [7]. Indeed, coronavirus (COVID-19) induced EFN has not been reported in the literature previously, but the mechanism underlying such inflammatory condition might be explained by the fact that epicardial adipose tissue (EAT), having the biggest proportions of lipogenesis and fatty acid metabolism among the visceral fat depots thus displaying thermogenic, mechanical, and metabolic properties, is a target for SARS-CoV-2 through ACE2 and the inflammatory cytokines interleukin-6 (IL-6), tumor necrosis factor- (TNF) α which are expressed at high levels across this tissue. Subsequently, such interaction promotes an augmented cascade of inflammatory processes leading to myocardial and surrounding structures complications including EFN [8].

Considering the clinical manifestation, EFN typically presents as chest discomfort in healthy individuals. It is a pleuritic pain, moderate to drastic in intensity, principally left-sided and some patients also experience shortness of breath on exertion. Our patient presented with the symptoms reported in the literature [9].

Physical examination is mostly unremarkable with normal laboratory results of creatine kinase and troponin [10]. Raised CRP levels were recorded in a few patients, probably due to the inflammatory process [11], which explains the lab value reported in the case we present. Electrocardiograms, as well as echocardiography, are non-specific in most cases [12]. EFN can be diagnosed by CT scan or CMR with characteristic imaging features [1].

Concerning its management, EFN had been treated primarily with thoracotomy and surgical excision in the past [13]. Nevertheless, due to the advancement in CT scan machines and their spatial resolution, a conservative approach toward management has been adopted, usually with non-steroidal anti-inflammatory medications for one to two weeks leading to symptom improvement [13]. And follow up by imaging after a few months to evaluate the resolution that has been proposed [1,14]. But, there resides a part for thoracotomy and surgery when the diagnosis remains skeptical and underlines a possibility of thoracic tumors.

## Conclusions

The list of differential diagnoses for COVID-19 patients presenting with chest pain to the ER department is long and ranges from EFN to myocardial infarction. EFN is an uncommon cause of acute chest pain. Yet, due to its benign, and self-limited course, it must not be overlooked in contrast to other life-threatening conditions.
